# Physician staffed helicopter emergency medical service dispatch via centralised control or directly by crew – case identification rates and effect on the Sydney paediatric trauma system

**DOI:** 10.1186/1757-7241-20-82

**Published:** 2012-12-18

**Authors:** Alan A Garner, Anna Lee, Andrew Weatherall

**Affiliations:** 1 CareFlight, PO Box 159, Barden St, Northmead, NSW 2145, Australia; 2 Department of Anaesthesia and Intensive Care, The Chinese University of Hong Kong, Prince of Wales Hospital, Shatin, NT, Hong Kong

**Keywords:** Paediatric, Trauma, Triage, Prehospital, Helicopter, Physician, Trauma centre

## Abstract

**Background:**

Severe paediatric trauma patients benefit from direct transport to dedicated Paediatric Trauma Centres (PTC). Parallel case identification systems utilising paramedics from a centralised dispatch centre versus the crew of a physician staffed Helicopter Emergency Medical Service (HEMS) allowed comparison of the two systems for case identification rates and subsequent timeliness of direct transfer to a PTC.

**Methods:**

Paediatric trauma patients over a two year period from the Sydney region with an Injury Severity Score (ISS) > 15 were retrospectively identified from a state wide trauma registry. Overall paediatric trauma system performance was assessed by comparisons of the availability of the physician staffed HEMS for patient characteristics, transport mode (direct versus indirect) and the times required for the patient to arrive at the paediatric trauma centre. The proportion of patients transported directly to a PTC was compared between the times that the HEMS service was available versus the time that it was unavailable to determine if the HEMS system altered the rate of direct transport to a PTC. Analysis of variance was used to compare the identifying systems for various patient characteristics when the HEMS was available.

**Results:**

Ninety nine cases met the inclusion criteria, 44 when the HEMS system was operational. Patients identified for physician response by the HEMS system were significantly different to those that were not identified with higher median ISS (25 vs 18, p = 0.011), and shorter times to PTC (67 vs 261mins, p = 0.015) and length of intensive care unit stays (2 vs 0 days, p = 0.045). Of the 44 cases, 21 were not identified, 3 were identified by the paramedic system and 20 were identified by the HEMS system, (*P* < 0.001). Direct transport to a PTC was more likely to occur when the HEMS dispatch system was available (RR 1.81, 95% CI 1.20-2.73). The median time (minutes) to arrival at the PTC was shorter when HEMS available (HEMS available 92, IQR 50-261 versus HEMS unavailable 296, IQR 84-583, P < 0.01).

**Conclusions:**

Physician staffed HEMS crew dispatch is significantly more likely to identify cases of severe paediatric trauma and is associated with a greater proportion of transports directly to a PTC and with faster times to arrival.

## Background

There is evidence that severely injured children have better outcomes if transported directly to a dedicated paediatric trauma centre (PTC) [1-5]. It is NSW state trauma policy that all severely injured children be managed in PTCs. Patients are either directly transported to a PTC by the EMS system or are transferred after initial assessment in an adult trauma centre (ATC). Prospective research from a single Sydney centre showed that the majority of severely injured paediatric trauma patients from within the Sydney region were initially taken to an ATC, requiring later secondary transfer to a PTC with an average delay of more than six hours from time of injury to arrival at the PTC [6]. More recent research indicates that this pattern persists, and that definitive care at an ATC was associated with the odds of death being between three and six times greater than for those receiving definitive care in a PTC [7].

The Head Injury Retrieval Trial (HIRT) [8] is a randomised controlled trial of physician prehospital care delivered via a helicopter emergency medical system (HEMS) compared with paramedic care for severe blunt head injury within the urban area of Sydney, Australia. Within the context of the HIRT, a system for screening the computerised dispatch system of the Ambulance Service of NSW (ASNSW) via a web link for patients likely to have a severe blunt head injury was instigated with screening carried out directly by the HIRT HEMS crew. Although children (age less than sixteen years) were excluded from the HIRT, the trial funder requested physician team response to children outside of the trial. Initially responses were mounted only to children likely to have a severe head injury but from May 2008, the dispatch criteria were expanded to include all types of severe paediatric injury and drownings.

In late 2007 the ASNSW partially replicated the HIRT dispatch system by introducing their own proactive tasking system to identify patients for physician prehospital response. This system consisted of a dedicated paramedic dispatcher, known as the Rapid Launch Trauma Coordinator (RLTC), who monitored the same screens as the trial HEMS team from a centralised control room. From May 2008 both the ASNSW RLTC and the HIRT HEMS crew monitored emergency calls utilising agreed criteria to activate the HIRT team for paediatric patients.

Although studies have been published examining criteria for activating HEMS for trauma there are very few studies that analyse the system of call screening, and none specifically in the paediatric population. The operation of the HIRT screening system for paediatric patients allowed a unique opportunity to compare two tasking systems for HEMS activation operating in parallel, as well as evaluating the effect of the HIRT model on the paediatric trauma system in greater Sydney.

The objective of this study was to compare the screening process used by the HIRT team versus RLTC in children with severe trauma for case identification rates, and subsequent timeliness and rate of direct transfer to a PTC.

## Methods

The study is a retrospective, registry based comparison of two case identification systems. The research question is for Injury Severity Score (ISS) > 15 children (under 16 years of age) who were notified to the public emergency services did use of screening process A - the HIRT team [intervention], compared to screening process B - the RLTC [comparator] result in:

• more patients being directly transferred from scene to a paediatric trauma centre and

• a shorter time to admission to the paediatric trauma centre.

Ethics committee approval was obtained from the Childrens Hospital at Westmead Human Research Ethics Committee. Cases were abstracted from the New South Wales (NSW) Institute of Trauma and Injury Management (ITIM) State trauma registry, Australia, if they met the following inclusion criteria:

o  Age < 16 years.

o  Incidents within the Sydney coordination area of the ASNSW.

o  Injury Severity Score (ISS) > 15.

o  Incident notification occurred via the 000 public access emergency call system.

o  Incidents occurred between 24^th^ May 2008 and 23^rd^ May 2010, i.e. the first 2 years of operation of the expanded dispatch criteria for paediatric trauma patients.

Cases transported to hospitals within the Sydney metropolitan area by private vehicle were excluded.

The HIRT service was available during daylight hours from May 2008 to September 2009. In September 2009 Night Vision Goggle technology was introduced to enable night responses. The hours of operation therefore changed to 10 am to 9:30 pm daily to match the peak hours of severe injury occurrence in the Sydney region. Reasons for HIRT service unavailability included prior tasking, case occurring outside the operational hours of the service or the service being off line due to helicopter maintenance or other issues.

The HIRT screening process utilised all four crew members (consultant grade doctor, paramedic, pilot and aircrewman) rotating hourly to monitor the screens. Cases which did not clearly match dispatch criteria were discussed amongst the team allowing simultaneous decision making input from both aviation and medical personnel. The RLTC system utilised a paramedic working either solo or paired with a second paramedic. Although the physician staffed HEMS service included a paramedic, the RLTC paramedics did not rotate to the HEMS service. The paramedics could seek advice directly from a consultant grade doctor rostered to the control room for part of the day or by phone at other times. Another physician staffed prehospital service is operated by the ASNSW in the Sydney region utilising road ambulances and helicopters. This service could be tasked by the RLTC either when the HIRT system was not available or in preference to the HIRT system. The RLTC system operated at all times when the HIRT system was functioning but does not operate 24 hours.

The dispatch systems operated in parallel looking for cases that met the agreed set of dispatch criteria. Criteria were:

All Severe Paediatric Trauma (<16 years) regardless of GCS

Including Severe

– Head injury.

– Truncal trauma.

– Limb injury.

– Penetrating injury.

– Near drownings.

– Burns especially airway.

– Multi Casualty Incidents where a child is likely to be involved.

Overall paediatric trauma system performance was assessed by comparisons of the availability of HIRT for patient characteristics, transport mode (direct versus indirect) and the times required for the patient to arrive at the paediatric trauma centre using Chi square test, t-tests or the Mann Whitney U test as appropriate. The proportion of patients transported directly to a PTC was compared between the times that the HIRT service was available (HIRT-A) versus the time that it was unavailable (HIRT-U) using a chi-square test to determine if the HIRT system altered the ratio of direct PTC transport to interhospital retrieval. A Kruskal-Wallis analysis of variance was used to compare the identifying systems for various patient characteristics when HIRT was available. Values are reported as mean and standard deviation (±SD), median and interquartile range (IQR), or as proportions (%). Relative risks (RR) and 95% confidence interval (95% CI) are reported. Statistical analysis was performed using SPSS version 18.0.3 (IBM).

## Results

Ninety nine cases met the inclusion criteria in the two year period. Forty four of these cases occurred during the HIRT-A periods and 55 occurred during the HIRT-U periods. Characteristics of patients in the two groups are presented in Table 1. There was no difference in the proportion of ICU admission between HIRT-A (61%) and HIRT-U (56%) groups (P = 0.62). Physiological data was not available from the ITIM trauma registry preventing more detailed comparisons of patient characteristics.

**Table 1:** Comparison of patient characteristics between the HIRT-A and HIRT-U periods

	**HIRT-A (n = 44)**	**HIRT-U (n = 55)**	***P value***
Mean Age - years	7.2 ± 5.1	5.9 ± 5.0	0.20
Mean ISS	23 ± 7.4	21 ± 4.9	0.13
Median length of ICU stay in days	3 (1-8)	2 (1-9)	0.74
Mechanism of injury			<0.01
Burn and/or scald	2 (5%)	10 (18%)	
Drowning	5 (11%)	3 (5%)	
Fall	13 (30%)	16 (29%)	
MVA	2 (5%)	8 (15%)	
Pedestrian	10 (23%)	5 (9%)	
Assault	0	7 (13%)	
Other	12 (27%)	6 (11%)	
Median length of hospital stay (days)	6.5 (1-29)	8 (5-20)	0.49

**Figure 1:**
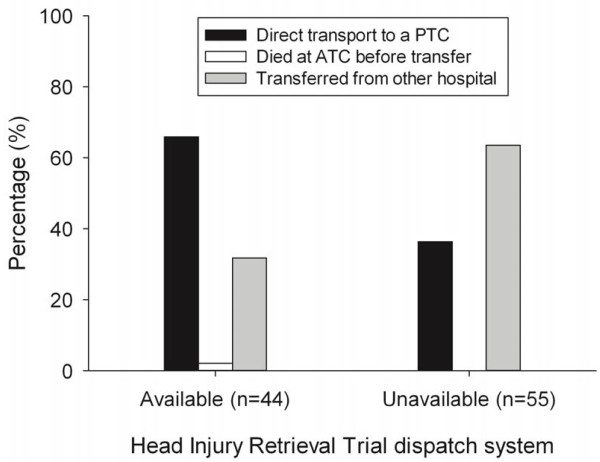
Proportion of patients (%) undergoing direct versus indirect transport to a PTC by availability of the HIRT dispatch system.

There was a significant difference in the proportion of cases that were transferred from other hospitals compared with direct transport to a PTC during the two periods in as indicated in Figure 1 (P < 0.01). Direct transport to a PTC was more likely to occur when the HIRT system was available than when it was not available (RR 1.81, 95% CI 1.20-2.73). One patient from the HIRT-A periods that was identified for physician response was in traumatic arrest at team contact, resuscitation was initiated and they were transported to the nearest ATC where they were subsequently pronounced deceased. All other severely injured paediatric patients who were taken to an ATC in the first instance were subsequently transferred to a PTC.

Excluding the child who died at the ATC before transfer, the median time (minutes) to arrival at the PTC was significantly shorter (P < 0.01) when the HIRT system was available (92, IQR 50-261) than when the HIRT system was unavailable (296, IQR 84-583). A subgroup analysis of the HIRT-A group shows a significantly shorter (P < 0.001) median time (minutes) to arrival at the PTC following direct transport (67, IQR 40-106) when compared to transport via an ATC (297, IQR 239-352).

There was a significant association between the system that identified the patient and HIRT availability (P < 0.001). Of the 44 cases occurring during the HIRT available periods, 21 were not identified for physician team response, 3 were identified by the RLTC and 20 were identified by the HIRT system, (*P* < 0.001 for the observed proportions if it is assumed that both dispatch systems are equally effective at identifying severe trauma cases). Of the 21 patients that were not identified for physician team response, road paramedics transported 10 to other hospitals, with eventual secondary transfer to a PTC at a later time. The remaining 11 patients were transported directly to a PTC. No patient that was not identified for physician response died of their injuries. The characteristics of the patients during HIRT-A periods by identification status are presented in Table 2. During the HIRT-A periods the HIRT system dispatched the HIRT team to 217 cases, 20 of which had ISS > 15 (9.2%), compared with the RLTC to 28 cases of which 2 had an ISS > 15 (7.1%) (P = 0.72). The RLTC also dispatched an ASNSW physician team to one case with ISS > 15. Data on the total number of ASNSW physician team dispatches to paediatric cases however was not available.

**Table 2:** Characteristics of patients identified by the various systems during the HIRT-A periods

	**Identified for physician team response by the HIRT system (n = 20)**	**Identified for physician team response by RLTC system (n = 3)**	**Not identified for physician team response (n = 21)**	***P***
Mean Age - years	7.3 ± 4.6	6.0 ± 4.6	7.3 ± 5.8	0.92
Median ISS	25 (18-34)	21 (18-no 75^th^ percentile available)	18 (17-23)	0.011
Median time to arrival in PTC - minutes	67 (31-101)	92 (20-no 75^th^ percentile available)	261 (68-348)	0.015
Median length of ICU stay - days	2 (0-11)	2 (0-no 75^th^ percentile available)	0 (0-2)	0.045
Median length of hospital stay - days	8 (1-42)	5 (1-no 75^th^ percentilile available)	7 (4-14)	0.83

### HIRT unavailable periods

Fifty five severe paediatric trauma cases occurred when HIRT was not available. Three cases were identified by the RLTC for physician response which were conducted by ASNSW physician teams. The RLTC is not a 24 hour system and data as to the operational status of the RLTC was not available to enable determination of the identification rate specifically during hours of RLTC only case identification. Further evaluation of these periods was therefore not possible.

## Discussion

The HIRT tasking system demonstrated significantly greater success in identifying severely injured paediatric patients for physician prehospital HEMS response and associated higher rates of direct transfer to the PTC when compared with the RLTC system. Although many trauma systems aim to keep total prehospital times to under sixty minutes, all patient groups exceeded this target, probably due to only two PTCs receiving patients from the relatively large geographic area of Sydney. However identification of trauma cases as appropriate for physician HEMS team response was associated with a significantly shorter time taken to reach the PTC. At times when the HIRT system was not available, direct transfer to PTC rates returned to the historical norm, with a corresponding increase in time taken to reach the PTC. The children that are identified for physician response in our system are significantly more severely injured than children that are not identified. No children died that were missed for physician dispatch by the combined HIRT/RLTC systems suggesting that the majority of critically injured children were identified.

As the dispatch systems operated in parallel with the first system to identify the case notifying the other, it is not possible to determine if cases that were identified by one system would eventually have been also identified by the other. The low rate of physician team dispatch during the HIRT-U periods when the RLTC operated in isolation suggests that most cases identified by the HIRT system would not have been identified by the RLTC. This could not be quantified however as data on the actual hours of operation of the RLTC in the HIRT-U periods was not available. Differences in injury mechanism between the HIRT-A and HIRT-U periods may also have contributed to this apparent difference.

Physician HEMS crew guided tasking offers several potential advantages compared with a paramedic dispatching from a centralised coordination centre. HEMS crew screening of emergency calls is undertaken by teams actively engaged in prehospital trauma care with a detailed understanding of both the aviation and medical capabilities of the HEMS team. It is possible that centralised screening by paramedics who are actively involved in the HEMS service may have produced better results. However involvement of the whole crew in screening activities allows up to date information about aviation conditions and response planning to proceed in parallel to the actual case identification process. Parallel processing has been shown to improve efficiency in other health care settings [9,10]. Well trained teams have also been demonstrated to almost always outperform the most proficient individual member of the team [11].

With the use of a team (HEMS crew), there is greater scope for managing fatigue related to the workload of case screening. By comparison, a system mostly reliant on an individual, as in the case of the RLTC, provides no mitigation for task-related fatigue or minimisation of inattention. Fatigue has been shown to significantly affect work performance in a variety of settings [12,13] as has inattention during constant vigilance tasks [14].

Definitive proof that physician crewed HEMS improves patient outcomes is not available. Concerns have been raised that physicians in the prehospital setting have little impact on mortality while potentially increasing scene time [15]. However, severely injured paediatric patients cared for by EMS paramedics are less likely to receive indicated interventions than adults with greater potential for complications associated with the interventions they do receive [16]. Prehospital care of paediatric patients by a physician has been shown to be associated with higher successful intervention rates [17]. A system that successfully detects severely injured children during the dispatch process, rapidly allocates advanced level providers and has a demonstrably shorter time to reach the PTC is likely to be optimal.

Limitations in the amount of information contained within the data set leave some questions unanswered. It is not possible to determine from the data set why some patients were identified for physician response and others were not. For example level of conscious has been demonstrated to be a strong predictor of severe injury in paediatric trauma patients [18,19]. Indications of decreased level of consciousness in the dispatching information may explain why only some patients were identified for physician response and why identified patients had higher ISS. Prehospital Glasgow Coma Scale scores were not recorded in the NSW ITIM Trauma Registry for the period of this study so that this possibility could not be explored. It is also possible that injuries to specific body regions may be more easily identifiable from the emergency call by dispatchers, but we were unable to identify this because the ISS is a composite score of injuries to all body regions.

There were significant differences in the mechanism of injury between the HIRT-A and HIRT-U periods. These differences may have been the result of diurnal variations in injury mechanism. It can be postulated that this affected the rate of direct transfer to a PTC although the mechanism for such an effect is unclear. If there is such an effect however it does not explain the difference in case identification rates between the HIRT and RLTC systems during the HIRT-A periods when both systems were operating in parallel.

The available data set also does not contain initial physiological data or long term patient outcomes. Prospective research incorporating long term patient follow-up would provide further information as to potential benefits of accurate dispatch triage, higher levels of prehospital care or rapid transfer to directly the PTC. Such prospective research may also be more likely to provide information as to which patients are most likely to benefit from early provision of high level care. Additionally, those transported by private vehicle direct to a hospital were excluded. It is likely that some of these patients would have suffered significant injuries and required secondary transfer to a PTC. Without accessing the EMS system however there is no way that they can be identified for physician response.

## Conclusion

Physician staffed HEMS crew screening and triage of emergency calls is significantly more effective at identifying cases of severe paediatric trauma than a centralised screening system staffed by paramedics who are not directly involved in provision of the HEMS service. This difference results in a higher rate of direct transfer to the PTC and faster times to arrival at the PTC. Further research is required to directly evaluate the impact on patient outcomes.

## Abbreviations

ASNSW: Ambulance service of New South Wales; ATC: Adult trauma centre; CI: Confidence interval; EMS: Emergency medical service; HEMS: Helicopter emergency medical service; HIRT: Head injury retrieval trial; HIRT-A: Head injury retrieval trial team available; HIRT-U: Head injury retrieval trial team unavailable; ICU: Intensive care unit; IQR: Interquartile range; ISS: Injury severity score; ITIM: Institute of trauma and injury management; NSW: New South Wales; PTC: Paediatric trauma centre; RLTC: Rapid launch trauma coordinator; RR: Relative risk; SD: Standard d-eviation.

## Competing interests

The authors declare that they have no competing interests.

## Authors’ contributions

AG conceived the study, participated in its design, and drafted the manuscript. AL participated in the design of the study, performed the statistical analysis and revised the manuscript. AW participated in the design of the study and revised the manuscript. All authors read and approved the final manuscript.
